# Assessing the construct validity of the Italian version of the EQ-5D: preliminary results from a cross-sectional study in North Italy

**DOI:** 10.1186/1477-7525-4-47

**Published:** 2006-08-10

**Authors:** Elena Savoia, Maria Pia Fantini, Pier Paolo Pandolfi, Laura Dallolio, Natalina Collina

**Affiliations:** 1 Department of Medicine and Public Health, Alma Mater Studiorum University of Bologna, Italy; 2 Azienda USL di Bologna, Bologna, Italy

## Abstract

**Background:**

Information on health related quality of life (HR-QOL) can be integrated with other classical health status indicators and be used to assist policy makers in resource allocation decisions. For this reason instruments such as the SF-12 and EQ-5D have been widely proposed as assessment tools to monitor changes in HR-QOL in general populations and very recently in general practice settings as well

**Aim:**

The primary goal of our study was to assess the construct validity of the Italian version of the EQ-5D in a general population of North Italy using socio-demographic factors and diagnostic sub-groups. Our secondary goal was to assess the concurrent validity of the EQ-5D and SF-12.

**Methods:**

The SF-12, the EQ-5D plus an additional questionnaire on socio-demographic characteristics, clinical conditions and symptoms were completed by 1,622 adults, randomly selected from the Registry of the Health Authorities of the city of Bologna, Italy. The primary care physician of each subject was contacted to report on the subject's health status.

**Results:**

Our findings indicate that the Italian version of the EQ-5D is well accepted by the general population (91% response rate), has good reliability (Cronbach's alpha 0.73), and shows evidence of construct validity.

**Conclusion:**

Our data provide a basis for further research to be conducted to assess the validity of the EQ-5D in Italy. In particular future studies should focus on assessing its ability to detect a clinically important change in health related quality of life over time (responsiveness).

## Background

Improving the health of local populations requires specific knowledge of the current levels of health status, which can be compared over time. However commissioning health care services carries with it the need to prioritize resources. For this reason policy makers have always expressed the necessity to identify variations within the communities they are serving, compare local data with normative population levels and eventually monitor changes in health status by diagnostic and socio-demographic sub-groups. Information on health related quality of life (HR-QOL) can be integrated with other classical health status indicators and be used to assist policy makers in resource allocation decisions [1-3]. For this reason instruments such as the SF-12 and the EQ-5D have been widely proposed as assessment tools to assess HR-QOL in general populations and very recently in general practice settings as well [4-9].

The SF-12 is a generic short form health survey, originally developed in the USA to provide a short alternative to the SF-36 [10]. It produces two summary measures evaluating physical and mental aspects of health derived from 12 questions. SF-12 has been successfully tested in several European countries, including Italy, on large samples of the general population, where it has proved its comprehensiveness, reliability, validity and cross-cultural applicability. [11]

The EQ-5D is an internationally developed health related quality of life measure that has been used throughout the world. [12] The main difference with the SF-12 is that the EQ-5D was developed as a preference-based measure, suitable for cost-effectiveness analysis. The most interesting characteristic of this instrument is the availability of a "utility index score" which for the decision makers following the principles of utilitarism makes the tool useful to set priorities in clinical settings and policy determinations. The utility view of quality of life refers to a subject's preference for a state of health. This view describes quality of life in a manner similar to the description of the benefits of a life insurance policy, where different monetary benefits are placed on the loss of various limbs. Although the EQ-5D has been extensively utilized in non-Italian settings, it lacks of empirical evaluations in Italy. The lack of information on the construct validity and reliability of the instrument as well as the absence of utilities estimated in the Italian population preclude its applicability.

The primary goal of our study was to assess the applicability, internal consistency, and construct validity of the Italian version of the EQ-5D in a random sample of the citizens of Bologna (North Italy). Our secondary goal was to test its concurrent validity with the SF-12.

## Methods

### Study population and data collection

A sample of 1,622 adults, aged 18–93, was randomly selected (simple random sample) from the Registry of the North and South Health Authorities of the city of Bologna, Italy. The adopted exclusion criteria were: people aged < 18 years, non residents of the two Health Authorities geographical areas, institutionalized subjects, and people not able to reason or understand and make decisions on their own. The study was performed in 2002 and the sample was expected to be representative of the residents of the geographical area covered by the two Health Authorities. A package with the SF-12 and the EQ-5D questionnaires plus an additional questionnaire on socio-demographic characteristics, clinical conditions and symptoms was sent home to the 1,622 subjects. The primary care physician of each subject was contacted by mail to report on the enrolled subject's health status by filling out a questionnaire to be returned to the Health Authority. In order to maximize the response rate each subject was contacted by telephone three times after the 7^th^, 14^th ^and the 21^st ^day from the inception of the survey. Delinquency after the third phone call resulted in dropping out the subject from the study and replacing her with a subject (same age and gender) randomly selected from the original sample. No reimbursement was offered to the study participants.

### Health status measurement

Two instruments were used to measure health related quality of life: the SF-12 v.1 and the EQ-5D. The SF-12 is a generic instrument that contains 12 items from the SF-36 Health Survey. The SF-12 estimates scale scores for four of the SF-36 eight health concepts (physical functioning, role-physical, role-emotional and mental health) using two items each; the remaining four health concepts (bodily pain, vitality, social functioning and general health) are each represented by a single item. We calculated the summary scores PCS-12 and MCS-12 using the scoring program described by Apolone [14].

The EQ-5D is a generic instrument, consisting of five three-level items, representing various aspects of health: mobility, self-care, usual activities, pain/discomfort and anxiety/depression (mood). Respondents can value their health in each domain by reporting whether they are experiencing none (score 1), some (score 2) or extreme (score 3) problems. These scores result in a health profile, e.g. a patient with profile 12113 has no problem with mobility, usual activities and pain/discomfort, some problems with self-care and extreme problems with anxiety/depression. Data of a visual analogue scale are also included in the EQ-5D and used by subjects to rate their health status between worst imaginable health state (score 0) to best imaginable health state (score 100). A utility index score was calculated for each subject's EQ-5D health status by applying the time trade-off-based valuations from a general UK population sample to the observed EQ-5D profile, as data from an Italian norm are not available at the present time. Using the data at hand self-rated index were also calculated using the EQ-VAS score method.

### Self-reported clinical conditions and socio-demographic data

In the package shipped to the subjects we also included an additional questionnaire to gather data on socio-demographic characteristics (gender, age, height, weight, level of education, occupation and marital status) and to investigate clinical conditions and/or symptoms that based on a literature search we hypothesized could affect everyday life (i.e. headache) and do not necessary require a medical consult or that are known to be reliable when self reported (i.e. diabetes, in treatment for dialysis). [17-21]

The self-reported questionnaire focused on the following symptoms or clinical conditions: visual impairment, hearing impairment, anxiety/depression, headache, diabetes, and dialysis. In addition a final open question was created asking the subject to report on other clinical conditions affecting her health status.

We used the level of education as proxy indicator of socio-economic status because information on income was not available. The level of education was described according to the Italian school system into 5 categories: less than elementary school degree, elementary school degree, middle school degree, high school degree, and college degree equivalent to less than 5 years of school, between 5 and 8, between 8 and 13 and more than 13 respectively.

We grouped the variable occupation into 7 categories: 1) managers, professionals, directors, businessmen, 2) public or private companies' employees, 3) labors, 4) house-keeping, 5) retirees, 6) students and 7) unemployed.

### Primary care physicians' assessments

The primary care physician of each subject was invited to give a clinical assessment on the enrolled subject. In order to gather such information in a structured and reliable way we designed a questionnaire including the definition of each investigated condition based on a review of the most recent clinical guidelines. References to the adopted guidelines were included and the questionnaire piloted tested before implementation. The clinical conditions included in the questionnaire were: hypertension, heart failure, angina, COPD, asthma, back-pain, cancer (diagnosed in the past 5 years), stroke, cirrhosis, arthritis (proved by X-ray documentation), myocardial infarction (occurred in the past 5 years), and stomach ulcer (proved by endoscopy).

### Construct and concurrent validity assessment

Construct validity refers to the evaluation of hypotheses about the expected performance of an instrument. A construct can be thought as a mini-theory to explain the relationships among attitudes, behaviors, and perceptions as well. Construct validation is an ongoing process of learning more about the construct, making new predictions and then testing them. It is a process where the theory and the measure are assessed at the same time [22]

Our approach in evaluating the EQ-5D construct validity was based on comparisons of mean value scores (for the EQ-5D index, EQ-self rated index and VAS) and ORs (for the EQ-5D items) across categories such as diagnostic or socio-demographic groups known or hypothesized to score differently "known group validity". For example we hypothesized that subjects of older age, with a lower educational attainment, female and unemployed scored lower compared to younger, more educated, male and employed subjects.

We also hypothesized that for the 14 identified diagnostic sub-groups scores would have been lower compared to healthy subjects.

The SF-12 was used to compare whether conceptually similar domains had higher correlations than conceptually unrelated domains.

### Data analysis

Internal consistency of the multi-item EQ-5D scale was calculated by means of Cronbach's α [22]. Average scores for the EQ-5D index (based on the UK population), EQ-self rated index, EQ-VAS, PCS-12 and MCS-12 scales were computed, as well as the proportions of respondents reporting impairment in the 5 EQ domains. The magnitude and significance of the ORs for the EQ-5D domains, as well as the sign and significance of the regression coefficients for the EQ-5D Index, EQ-self rated index, EQ-VAS, PCS-12 and MCS-12 scores were used as discriminative measurement tools in testing "known group validity". The level of significance was set at 0.05. When the assumption of linearity was satisfied we adjusted the sub-groups mean scores for age and/or gender using linear regression. We used logistic regression to adjust when dealing with categorical variables. Adjustment was performed because age and gender are known to be associated with both scores of health status and particular socio-demographic and clinical variables. Therefore, considered as potential confounders. The effect of self-reported health problems and of the physicians' reported diagnosis on the EQ-5D dimensions was estimated using logistic regression while the effect of the same variables on the EQ-5D index, EQ-self rated index, EQ-VAS, PCS-12 and MCS-12 was estimated using multivariate linear regression.

The concurrent validity of the EQ-5D and SF-12 in this respondent sample was tested examining the relationship between the self-reported EQ-5D and the SF-12 component scores. The relationships between comparable dimensions and component scores, such as anxiety/depression with the MCS-12 and mobility, self-care, usual activities and pain/discomfort with the PCS-12 were hypothesized to be stronger than between less comparable dimensions and component scores, for example mobility and the MCS-12. In contrast the EQ-VAS score was expected to correlate reasonably well with both the MCS-12 and PCS-12. The correlation between the EQ-index (calculated on the UK population time trade off criteria) and the EQ-self rated index was also computed. The strength of the correlation was determined by Cohen's (1992) criteria where large correlations are described as being >0.50, medium correlations range between 0.30–0.49 and small correlations range between 0.10–0.29.

The compare the "discriminant" validity of the two questionnaires we used the magnitude of ratio of the F-test from multivariable analyses of variance. We hypothesized the ratio to be greater for comparable dimensions such as PCS-12 and the 4 EQ-functional dimensions compared to non-comparable dimensions such as PCS-12 and the anxiety dimension.

Data were analyzed using Statistical Package for the Social Sciences (SPSS) version 11.5.

## Results

### Response rates

Completed questionnaires were collected from 1,555 subjects, 96% response rate. Of the 1,555 subjects 1,421 (91%) completed the EQ-5D, 1,326 (85%) the EQ-VAS, and 1,364 (88%) the SF-12.

Considering the original sample 16.4% of non-respondents were replaced. Thirty-six percent (524) of respondents that completed all items of the EQ-5D reported no problems (i.e. 11111) on all five dimensions. Of the 243 possible health states described by the EQ-5D, respondents reported 47 different health states. Therefore the ceiling effect of the EQ-5D was modest compared to other studies [23].

### Demographics of participants

The subject socio-demographic information is presented in Table 1. The mean age (SD) of participants was 50.23 (18.13) years and ranged from 18 to 93, 52% were female. More than half of subjects (60%) reported to have achieved a middle school educational level. The most frequent job position was public employee (21% of the total sample) while 28% of participants were retirees. Most participants were married (62%).

**Table 1:** Socio-demographic variables.

**Characteristic**	**N**	**%**
**Age (years)**		
18–24	95	6.3
25–34	268	17.9
35–44	273	18.2
45–54	254	17.0
55–64	229	15.3
65–74	205	13.7
> 75	173	11.6
**Gender**		
Male	718	48
Female	779	52
**Education**		
< 5 years	49	3
5 years	435	29
8 years	402	27
13 years	490	33
> 13 years	104	7
**Occupation**		
Manager/Director	32	2
Professional	80	6
Business man	179	12
Employee	305	21
Labors	193	13
House keeping	169	12
Student	44	3
Retired	410	28
Unemployed	25	2
**Marital status**		
Single	380	25.5
Married	930	62.4
Widowed	149	10.0
Divorced/Separated	31	2.1

### EQ-5D reliability and construct validity

Cronbach's coefficient α was 0.73 showing good reliability of the instrument.

The mean EQ-5D index score (SD) was 0.81 (0.22) and the mean EQ-VAS score (SD) was 77.0 (17.4). The EQ-VAS sample mean score of 77.0 (17.4) was lower than the general population norm of 82.5 (17) from the U.K. sample. [24]The Pearson correlation coefficient between the EQ index and EQ-VAS was 0.65 (p < 0.001) and between the EQ index and EQ-self rated index was 0.89 (p < 0.001). With the exception of the age category 25–34, mean scores on both the EQ index and EQ-VAS decreased with increasing category of age (Table 2). Age and gender resulted to be determinants of the outcome "reporting some or extreme problems" in each of the 5 dimensions of the EQ with seniors and female reporting lower scores (Table 2). The adopted proxy indicator of socio-economic status (educational level) was related to the presence of severe or moderate symptoms in the 5 dimensions of the EQ, and low scores in the EQ-index and EQ-VAS, even after adjusting for age and gender simultaneously. Therefore socio-economic status was negatively related to quality of life. Among the different marital status widowed showed the highest significantly different percentage of reported problems on the EQ dimensions with the exception of anxiety and depression, low scores were reported in the EQ index and EQ-VAS as well, always adjusting for age and gender. We did not find a linear relationship between occupational status and quality of life. However we demonstrated a difference in quality of life in the mean scores ANOVA *F-test *(p < 0.001) after adjusting for age and gender. Among occupations, retirees reported the lowest scores.

**Table 2:** Responses to the EQ-5D and SF-12 by socio-demographic characteristics.

Variable	**% Reporting a problem (moderate or severe) for categorical variables or mean value for numeric variables.**								
	**PCS**	**MCS**	**Mobility**	**Self-care**	**Usual activities**	**Pain/discomfort**	**Anxiety/depression**	**EQ-5D**_**index**_	**EQ-5D**_**vas**_

Total sample	48.4	47.7	13.6	5.8	14.1	45.4	39.3	0.8	77.0
**Age**^**α**^	*	**	*	*	*	*	*	*	*
18–24	50.3	48.5	4.3	3.2	4.3	30.1	44.1	0.8	81
25–34	50.8	47.7	7.6	3.8	10.3	40.2	38.3	0.8	80
35–44	50.3	48.5	10.4	5.4	10.3	42.6	32.0	0.8	79
45–54	49.2	48.0	7.3	2.4	9.7	45.7	40.0	0.8	80
55–64	48.2	47.6	14.7	6.7	17.6	53.6	50.7	0.8	76
65–74	46.1	47.4	21.2	2.6	18.2	59.6	44.7	0.8	74
> 75	42.4	45.3	40.0	21.8	37.3	67.3	53.3	0.7	68
**Gender**^β^	*	**	*	*	*	*	*	*	*
Male	50.0	49.5	9.6	4.3	10.1	40.8	31.9	0.8	80
Female	46.8	45.8	19.2	8.0	19.8	56.2	51.9	0.8	75
**Occupation**^δ^	*	*	*	*	*	*	*		
1	54.6	42.5	4.0	0	8.0	32.0	48.0	0.9	75
2	43.7	46.2	29.3	13.2	26.6	64.6	52.5	0.7	70
3	50.3	50.3	4	1.1	7.0	43.0	37.1	0.9	82
4	43.7	45.1	21.0	20.7	19.1	16.8	16.5	0.7	70
5	53.9	46.5	0.5	-	1.0	1.3	2.4	0.9	85
6	42.5	46.6	64.9	71.3	56.9	39.1	34.5	0.7	67
7	54.4	42.5	0.5	-	1.0	1.2	2.0	0.9	75
**>Educational level γ**	*	*	*	*	*	*	*	*	*
< 5 years	36.8	41.4	50.0	29.8	48.9	87.5	60.4	0.55	60.7
5 years	43.3	46.5	30.9	12.8	27.6	65.1	49.5	0.72	68.7
8 years	49.4	48.5	8.2	3.3	11.5	49.2	45.4	0.82	79.2
13 years	51.8	48.4	4.2	1.5	5.8	33.3	32.4	0.88	82.6
> 13 years	52.9	48.1	2.0	1.0	2.9	32.7	38.2	0.88	83.2
**Marital status**									
Single^γ^	52.5*	49.0	6.6*	5.6**	11.0**	15.7*	19.2**	0.88*	82.9*
Married^γ^	48.0	47.6	60.2	52.8	60.7	67.8*	65.5*	0.81	77.0
Widowed^γ^	39.1*	44.3**	31.3**	39.3**	26.5**	14.6**	13.0	0.61*	63.2*
Divorced^γ^	51.5**	47.7	1.9	2.2	1.8	2.0	2.3	0.80	81.2

^α^adjusted for gender; ^β^adjusted for age; ^γ^adjusted for age and gender; *p <0.001 **p <*0.05*

^δ^1 = manager, director, business-man; 2 = public employee; 3 = labor, 4 = house keeping, 5 = student, 6 = retiree, 7 = unemployed.

With respect to the clinical conditions referred by the patient all were significantly associated with increased odds of reporting impairment in all 5 EQ dimensions. Results are displayed in table 3. In particular visual impairment and hearing impairment were the ones with the greatest impact on mobility, self-care and usual activities. For subjects affected by visual impairment compared to subjects not affected by the clinical condition we obtained a 600% increased odds of reporting impairment in the mobility domain (OR = 7.0, 95% C.I. 4.7–10.4), a 690% increased odds of reporting impairment in the self care domain (OR = 7.9 95% C.I. 4.8–12.9) and a 640% increased odds of reporting impairment in the usual activities domain (OR = 7.4, 95% C.I. 5.0–10.9). Visual impairment was asked as a persistent condition not solved by the use of glasses. For subjects affected by hearing impairment compared to subjects not affected by the clinical condition we obtained a 480% increased odds of reporting impairment in the mobility domain (OR = 5.8, 95% C.I. 4.0–8.3), a 430% increased odds in the self-care domain, and a 370% increased odds in the usual activities domain (OR = 4.7, 95% C.I. 3.3–6.7). Having a headache at least once a week affected mainly the pain and discomfort domain with a 300% increased odds of reporting impairment compared to subjects not affected by the clinical condition (OR = 4.0, 95% C.I. 3.0–5.4)

**Table 3:** Responses to the EQ-5D and SF-12 by clinical condition.

**Clinical Condition (n)**	**OR of reporting an impairment**					**Beta coefficient**		
	**Mobility**^α^	**Self-care**^α^	**Usual activities**^α^	**Pain/discomfort**^α^	**Anxiety/depression**^α^	**EQ-5D**_**index α-UK**_	**EQ-5D**_**vas**_^α^	**EQ-VAS score**

Diabetes (59)	4.8 (2.7–8.7)	4.8 (2.4–9.5)	3.5 (1.9–6.2)	2.8 (1.5–5.2)	1.7 (1.0–3.0)	-0.11*	-0.19*	-0.13*
Without (1423)								
Visual impairment (159)	7.0 (4.7–10.4)	7.9 (4.8–12.9)	7.4 (5.0–10.9)	4.0 (2.6–6.1)	2.6 (1.8–3.7)	-0.32*	-0.34*	-0.34*
Without (1295)								
Hearing impairment (204)	5.8 (4.0–8.3)	5.3 (3.3–8.4)	4.7 (3.3–6.7)	3.3 (2.3–4.6)	2.1 (1.5–2.9)	-0.24*	-0.24*	-0.25*
Without (1275)								
Anxiety (233)	2.5 (1.7–3.6)	3.2 (2.0–5.2)	2.9 (2.1–4.2)	2.0 (1.5–2.7)	17.1 (10.9–26.9)	-0.31*	-0.26*	-0.29*
Without (1234)								
Head ache at least once a week (351)	1.7 (1.2–2.3)	1.6 (1.0–2.6)	1.9 (1.4–2.7)	4.0 (3.0–5.4)	2.4 (1.8–3.1)	-0.24*	-0.13*	-0.20*
Without (1118)								
Hypertension (277)	4.0 (2.8–5.8)	4.6 (2.7–7.7)	2.9 (2.0–4.2)	1.8 (1.3–2.5)	1.5 (1.1–2.0)	-0.19*	-0.29*	-0.20*
Without (875)								
Heart failure (35)	22.0 (8.6–56.0)	21.0 (9.4–46.3)	14.1 (6.0–33.2)	4.3 (1.6–11.5)	2.2 (0.9–4.9)	-0.25*	-0.23*	-0.25*
Without (1125)								
Angina (21)	3.3 (1.2–8.6)	4.5 (1.5–13.4)	5.3 (2.1–13.6)	1.9 (0.7–5.3)	3.1 (1.1–8.3)	-0.09**	-0.11**	-0.10**
Without (1140)								
COPD (84)	4.4 (2.6–7.5)	3.0 (1.5–6.0)	4.8 (2.8–8.2)	3.3 (1.9–5.8)	1.2 (0.7–2.0)	-0.14*	-0.21*	-0.19*
Without (1075)								
Asthma (39)	1.3 (0.5–3.0)	2.4 (0.9–6.5)	3.1 (1.5–6.5)	1.2 (0.6–2.5)	1.6 (0.8–3.2)	-0.06**	-0.07**	-0.07**
Without (1140)								
Back pain (327)	4.6 (3.2–6.7)	3.0 (1.8–5.1)	2.9 (2.1–4.2)	3.6 (2.7–4.9)	1.5 (1.1–1.9)	-0.25*	-0.28*	-0.25*
Without (814)								
Stomach ulcer (35)	1.9 (0.8–4.3)	2.0 (0.7–5.8)	2.9 (1.4–6.3)	3.6 (1.5–8.6)	1.6 (0.8–3.3)	-0.12*	-0.08**	-0.10*
Without (1120)								
Arthritis (314)	7.8 (5.3–11.6)	3.7 (2.2–6.2)	4.1 (2.8–5.8)	4.5 (3.3–6.3)	1.5 (1.1–2.0)	-0.3*	-0.35*	-0.32*
Without (833)								
Obesity (BMI >30) (163)	3.5 (2.3–5.2)	1.9 (1.1–3.4)	2.2 (1.4–3.3)	1.7 (1.2–2.4)	1.3 (0.9–1.8)	-0.08**	-0.12*	-0.11*
Without (1283)								

^α^Adjusted for age and gender where * *p *<*0.001 *and ** *p *<*0.05*

Subjects reporting to be affected by anxiety and to be in treatment for the condition were 17.1 times more likely to report impairment in the anxiety and depression domain compared to subjects not affected by the condition (OR = 17.1, 95% C.I. 10.9–26.9)

Diabetes was associated to all 5 dimensions with ORs ranging from 4.8 (95% C.I. 2.7–8.7) in the mobility domain to 1.7 (95% C.I. 1.0–3.0) in the anxiety and depression domain.

With respect to the clinical conditions referred by the subject's primary care physician all were significantly associated with increased odds of reporting impairment in all 5 EQ dimensions. Table 3. Most of them were strongly associated to increased odds of reporting impairment in the mobility domain. In particular heart failure is the one showing the greatest odds (OR = 22.0, 95% C.I. 8.6–56.0). But also arthritis, back pain, COPD, and obesity (BMI>30) were strongly associated to mobility impairment. Angina, asthma and COPD mainly affected the usual activities domain. Angina was associated with the anxiety and depression domain with 210% increased odds of reporting impairment (OR = 3.1, 95% C.I. 1.1–8.3) compared to subjects not affected by the clinical condition. Stomach ulcer was mainly associated with the pain and discomfort domain with 260% increased odds of reporting impairment (OR = 3.6, 95% C.I. 1.5–8.6) compared to subjects not affected by this condition.

All clinical conditions showed a negative impact on HR-QOL when the EQ-5D index, EQ-self rated index and the EQ-VAS scores were taken into considerations. Applying a linear regression model, adjusting for age and gender, regression coefficients ranged from – 0.08 (p < 0.005) for obesity and stomach ulcer impacting the EQ-5D index and EQ-5D VAS score respectively and -0.35 (p < 0.001) for arthritis impacting the EQ-VAS score, results are shown on Table 3.

### EQ-5D and SF12 concurrent validity

As expected the relationships were stronger between the EQ-5D functional dimensions and the PCS-12, and between the MCS-12 and the anxiety/depression dimension. As a matter of fact the correlation coefficients between PCS-12 and the functional dimensions ranged from 0.65 for the usual activities domain to 0.43 for the self-care domain. As expected the MCS-12 score well correlated with the anxiety and depression domain r = 0.59. The relationships between the less comparable dimensions and the component scores were not as strong. In fact the correlation coefficients between the MCS-12 and the physical items ranged from 0.34 for the usual activities domain to 0.25 for the self-care and mobility domains. While the PCS-12 score correlated with the anxiety and depression domain with a coefficient as low as of 0.29. The EQ-VAS scores were positively correlated with both component scores; r = 0.46 for MCS-12 and r = 0.66 for PCS-12. All correlations were significant with p-value < 0.001.

In addition corresponding dimensions and summary scores were more strongly related (eg, mobility and PCS-12; F ratio = 401.45, p-value < 0.001, or anxiety and MCS; F ratio = 356.8, p-value < 0.001) than dissimilar dimensions (eg, mobility and MCS-12; F ratio = 46.8 or anxiety and PCS-12 F ratio = 63.6, p-value < 0.001).

## Discussion

In this study we investigated the construct validity of the Italian version of the EQ-5D administering the instrument to a sample of citizens living in Bologna (North Italy). We provided evidence supporting the construct validity and reliability of the instrument supported by data on socio-demographic characteristics and diagnostic sub-groups of the participants. Strength of our study was the achieved high response rate and the primary care physicians' support in assessing each subject's health status.

The instrument resulted to be consistent with the hypothesized construct and showed good reliability. The convergent and discriminant validity of the EQ-5D were also supported by the relationship with the SF-12 component scores observed in the data, with stronger relationships observed between the PCS-12 scores and the functional dimensions than with the anxiety/depression dimension. Likewise the MCS-12 scores differentiated the level of anxiety/depression dimension more strongly than for the levels of the functional dimensions of mobility, self-care, usual activities and pain/discomfort.

We consider our results as a preliminary step towards the empirical validation process of the EQ-5D in Italy. However some limits of our research should be taken into consideration.

Our sample was representative of two health district areas of the city of Bologna, the Italian territory is extremely heterogeneous in terms of population characteristics such as age, socio-economic status, health status and life-style. In particular differences are present in most health indicators between the North and South of the country. Therefore any inference on the Italian population should be cautious. The utility value calculated for the EQ-5D was based on the U.K. population norm data, debate on the cross adaptability of such scores has not been solved yet. The absence of values based on the Italian population affects the most important characteristic of the instrument, which is its use in cost-effectiveness analysis. However EQ-self rated index scores were derived and showed a high correlation with the UK EQ-index scores.

A known limit of the EQ-5D is to have a 3 responses format, as a consequence subject to a considerable ceiling effect. However in our sample it appeared that the dimensions were discriminative enough to distinguish between respondents with and without specific clinical conditions.

An other limit of our study was not being able to assess the instrument's responsiveness, which is extremely important for its use in monitoring a population's health status.

## Conclusion

Our data provide evidence on the construct validity of the Italian version of the EQ-5D in a general population of a large city in North Italy. The measurements of the EQ-5D behaved in patterns that were consistent with recognized socio-demographic differences in health status.

Future studies should focus on assessing the instrument's ability to detect a clinically important change in health related quality of life over time (responsiveness) in order to be able to adopt the tool to monitor a population's health status. However in addition to a psychometric approach measurement/metric equivalence of the Italian version of the EQ-5D should also be investigated. In particular the clinically minimal important difference (MCID), which is defined as the smallest difference between the scores in a questionnaire that the patient perceives to be beneficial should be assessed in an Italian sample should be assessed. A national effort in designing a study with a representative sample of the Italian population will be a necessary step to increase evidence on the EQ-5D applicability in Italy.

## Competing interests

The author(s) declare no competing interest in the conduction of the study.

## Authors' contributions

Dr. Pandolfi designed and led the study, Dr. Collina led the implementation of the study in the health district under her authority, Dr. Fantini offered methodological support in designing the study and for the data management plan, Dr. Dallolio coordinated data entry and data analysis, Dr. Savoia provided assistance with data analysis and the narrative of the manuscript. All authors revised the text and provided information and comments to its final version.
